# Efficacy of a novel topical combination of esafoxolaner, eprinomectin and praziquantel against *Rhipicephalus sanguineus* in cats

**DOI:** 10.1051/parasite/2021020

**Published:** 2021-04-02

**Authors:** Eric Tielemans, Anthony Pfefferkorn, Alta Viljoen

**Affiliations:** 1 Boehringer-Ingelheim Animal Health 29 avenue Tony Garnier 69007 Lyon France; 2 Clinvet International (Pty) Ltd. P.O. Box 11186 Universitas Bloemfontein 9321 Republic of South Africa

**Keywords:** Cat, Esafoxolaner, Tick, *Rhipicephalus sanguineus*, Efficacy

## Abstract

Esafoxolaner is a purified enantiomer of afoxolaner with insecticidal and acaricidal properties. It is combined with eprinomectin and praziquantel in a novel topical endectoparasiticide formulation for cats. The efficacy of this novel formulation was assessed in an experimental study against induced infestation of *Rhipicephalus sanguineus* ticks. Twenty cats were randomly allocated to either a placebo control group or a treated group in a 1:1 ratio. Infested cats were treated topically once at the minimum recommended dose. The study was designed to assess curative efficacy 48 h after treatment and to test preventive efficacy 48 h after weekly infestations for 2 months. At each weekly infestation, all cats were infested with 25 male and 25 unfed female *R. sanguineus* ticks. At each tick count, at least 6 in 10 control cats had a retention of 13 (26%) or more live ticks, demonstrating adequate infestation throughout the study. Curative efficacy on existing tick infestation was 90%; preventive efficacy over the following 6 weeks was at least 96%.

## Abstract (fr)

L'esafoxolaner est un énantiomère purifié d’afoxolaner, aux propriétés insecticides et acaricides. Il est combiné à éprinomectine et praziquantel dans une nouvelle formulation topique endectoparasiticide pour chats. L’efficacité de cette nouvelle formulation a été testée lors d’une étude contre des infestations expérimentales avec des tiques *Rhipicephalus sanguineus.* Vingt chats ont été répartis au hasard soit dans un groupe témoin placebo soit dans un groupe traité (rapport 1:1). Les chats infestés ont été traités par voie topique une fois à la dose minimale recommandée. L’étude a été conçue pour une évaluation de l’efficacité curative 48 heures après traitement et pour des évaluations d’efficacité préventive 48 heures après chaque infestation hebdomadaire pendant 2 mois. À chaque infestation hebdomadaire, tous les chats étaient infestés par 25 mâles et 25 femelles de *R. sanguineus*, non nourris. À chaque comptage, au moins 6 chats sur 10 du groupe placebo contrôle étaient infestés avec au moins 13 (26 %) tiques vivantes, ce qui a validé le modèle d’infestation. L’efficacité curative sur tiques présentes avant traitement a été de 90 %, l’efficacité préventive durant les six semaines suivantes a été d’au moins 96 %.

## Introduction

*Rhipicephalus sanguineus*, the brown dog tick is the most widespread tick species in the world. It affects dogs living in urban and rural areas, and throughout the year [5, 10]. The main host of *R. sanguineus* is the dog, on which it can complete its full development cycle, but this species also infests other mammals, including cats, rodents, livestock and humans at immature or adult stages [5, 8, 9]. *Rhipicephalus sanguineus* is an important vector of a diverse range of pathogens, such as *Babesia, Cercopithifilaria, Hepatozoon, Ehrlichia*, and *Rickettsia* [1–5, 9, 14, 18]. *Rhipicephalus sanguineus* is highly adapted to warm conditions in tropical and subtropical climates, either dry or wet, but can also thrive in temperate climates where global warming contributes to its increased presence and relevance from a public health perspective [5]. *Rhipicephalus sanguineus* is not a very common tick species infesting cats, like *Ixodes* spp. or *Amblyomma americanum*; however, it is occasionally described on cats, especially in the presence of dogs [5, 16, 17]. The behavior of free-roaming cats including predation of infested rodents, access to hidden places where immature *R. sanguineus* stages moult, and movements may expose cats to immature *R. sanguineus* infestation [11]. Furthermore, the grooming habits of cats allow them to remove larger adult ticks more easily than small larvae or nymphs, which might be another explanation as to why a larger proportion of immature stages than adults was found on cats [11].

A novel topical combination of esafoxolaner, eprinomectin and praziquantel was developed to offer a wide parasiticide spectrum and integrated control of cat parasites. Afoxolaner has already been proven effective against *R. sanguineus* in dogs [12]. Afoxolaner is a racemic mixture, and esafoxolaner is the active purified enantiomer. This article describes a study performed to evaluate the efficacy of this novel formulation for the treatment and control of *R. sanguineus* infestations in cats.

## Materials and methods

### Study design

The study was designed in accordance with the “World Association for the Advancement of Veterinary Parasitology (W.A.A.V.P.) guidelines for evaluating the efficacy of parasiticides for the treatment, prevention and control of flea and tick infestation on dogs and cats” [13]. It was conducted in accordance with Good Clinical Practices as described in International Cooperation on Harmonization of Technical Requirements for Registration of Veterinary Medicinal Products (VICH) guideline GL9. The study protocol had been reviewed and approved by the Sponsor’s and local Institutional Animal Care and Use Committees. Cats were handled with due regard for their wellbeing.

The study was conducted under a negative-controlled and randomized design. Randomization was based on a pre-treatment live *R. sanguineus* count, 48 h after infestation. The efficacy assessment was based on comparison of live ticks found in the control and treated groups at identical weekly time-points after treatment.

The study was performed in South Africa, during October–December 2018. All personnel collecting animal health and efficacy data were blinded to treatment. Cats were single housed during the study to avoid inter-animal treatment contamination.

### Animals

Twenty purpose-bred, healthy laboratory shorthair cats, 10 males and 10 females, aged 6 months–5 years old and weighing 1.9–5.3 kg were included in the study.

### *Rhipicephalus sanguineus* strain

The *R. sanguineus* originated from the field in 2011 in Georgia, United States. The colony had been maintained at the test facility since 2014 on rabbits not treated with acaricides.

### Treatment

Cats were treated once on Day 0. The treatment was applied topically on the skin, after parting the hair, on one spot in the midline of the neck between the base of the skull and the shoulder blades. Cats assigned to the placebo control group were treated with mineral oil at 0.12 mL/kg, cats assigned to the treated group were applied the novel formulation at the minimum recommended dose of 0.12 mL/kg, delivering 1.44 mg/kg esafoxolaner, 0.48 mg/kg eprinomectin, and 10.0 mg/kg praziquantel.

To detect the presence or absence of any treatment-related or unrelated health abnormality, health observations were conducted daily and at hourly intervals for 4 h after treatment.

### Tick infestations

Each cat was infested in a random order, on Days – 2, 7, 14, 21, 30, 37, 44, 53 and 58. Animals were sedated and placed in an infestation chamber equipped with adhesive tapes at the edges to act as a tick barrier. Once sedated, cats were equipped with an Elizabethan collar to limit grooming. Twenty-five female and 25 male *R. sanguineus* were then placed on the lateral side of each cat and care was taken to avoid the treatment application site. After a maximum of 4 h, cats were removed from the crates and the remaining free ticks placed back on the cat. The Elizabethan collar was left until the tick count procedure.

### Tick counts

Tick counts were performed in a random order. Attached and unattached live and dead ticks were removed and counted approximately 48 h after treatment on Day 2, and approximately 48 h after the subsequent infestations on Days 9, 16, 23, 32, 39, 46, 55 and 60. Tick counts were performed by parting and feeling through the cat’s hair with finger tips. When a tick was detected, the hair was further parted, visual confirmation of the tick’s presence was made, the tick was removed, and the live or dead status of the tick confirmed. After an area was cleared by this method, the area was combed using a fine-toothed flea comb for a second check for tick presence. Each tick was observed for signs of viability or mortality. Viability was evaluated if necessary by breathing on the motionless tick and observing the presence or absence of reaction to this stimulation. Protective clothing (e.g. gowns/coats, gloves, etc.) and combs were changed between each cat to prevent cross-contamination.

## Statistical analysis

To compute the percent efficacy, the arithmetic mean of the live tick counts was calculated by group at each time-point. Percent efficacy of the treated group with respect to the control group was calculated using the formula [(*C* − *T*)/*C*] × 100, where *C* = arithmetic mean for the control group, and *T* = arithmetic mean for the treated group. The log-count of each treated group was compared to the log-count of the control group using an *F*-test adjusted for the allocation blocks used to randomize the animals to the treatment groups at each time-point separately. The mixed procedure in SAS version 9.4 was used for the analysis, with group listed as a fixed effect and the allocation blocks listed as a random effect.

## Results

The individual retention of live ticks on the control cats was 18 ticks on average and ranged from 3 to 38 ticks. At each tick count time-point, at least 6 in 10 control cats had a retention of 13 (26%) or more live ticks, which is considered in the WAAVP guideline [13] as demonstrating adequate infestation throughout the study and vigorous tick population.

The curative efficacy of one application of the novel formulation on existing tick infestation was 90%, the preventive efficacy over the following 6 weeks was at least 96% (Table 1).

**Table 1:** *Rhipicephalus sanguineus*, mean live tick counts per group and efficacy results.

		*n*^5^	Day 2	Day 9	Day 16	Day 23	Day 32	Day 39	Day 46	Day 55	Day 60
Control group^1^	AM^3^	10	15.7	19.5	21.9	18.5	19.2	15.7	17.9	16.9	14.2
Treated group^2^	AM^3^	10	1.6	0.0	0.0	0.0	0.2	0.5	0.7	2.0	5.3
		% Efficacy^4^	89.8	100.0	100.0	100.0	99.0	96.8	96.1	88.2	62.7
		*p*-value	<0.0001	<0.0001	<0.0001	<0.0001	<0.0001	<0.0001	<0.0001	<0.0001	0.0191

1 The Control (CP) group was treated topically once on Day 0 with 0.12 mL/kg Technical oil.

2 The Treated (IVP) group was treated topically once on Day 0 with 0.12 mL/kg of the novel formulation of (S)afoxolaner, eprinomectin and praziquantel.

3 Percent efficacy = [(*C* − *T*)/*C*] × 100, where T and C are arithmetic means (AM) of the IVP group and the CP group, respectively.

4 *p*-value = two-sided *p*-value from the MIXED model of the IVP group and the CP group.

5 *n* = number of cats per group: one cat was removed on Day 50 because deemed unsuitable to remain in the study for excessive tick bite wounds, consequently *n* = 9 in the control group on Days 55 and 60.

No adverse reactions related to treatment were observed.

## Discussion and conclusion

The results of this study illustrate the high level of efficacy of the novel topical formulation of esafoxolaner, eprinomectin and praziquantel against *R. sanguineus* infestation in cats for curative efficacy of existing infestation within 48 h of the treatment application, and for preventive efficacy within 48 h of new infestations for 6 weeks.

Even though *R. sanguineus* is not considered a major tick species of cats, it should not be underestimated, in view of its potential contribution to dog infestation by free roaming and spreading of immature and adult stages, and severe vector-borne pathogen transmission to dogs and also humans.

This novel association of esafoxolaner, eprinomectin and praziquantel offers a broad spectrum of efficacy against the main parasites of cats including ecto- and endoparasites. The control of multiple and various concurrent parasitic infestations by a range of cat parasites is important for cats but also public health [4, 6, 7, 15].

Alongside a high level of efficacy and safety, owner and cat compliance is an important feature of success for this type of therapeutic approach, and the easy conditions of use and of treatment application of this product are necessary to guarantee a high level of compliance.

## Conflict of interest

The work reported herein was funded by Boehringer-Ingelheim. Eric Tielemans and Anthony Pfefferkorn are current employees of Boehringer-Ingelheim, Alta Viljoen is a current employee of Clinvet International. Other than that, the authors declare no conflict of interest. This document is provided for scientific purposes only. Any reference to a brand or trademark herein is for information purposes only and is not intended for any commercial purposes or to dilute the rights of the respective owners of the brand(s) or trademark(s).
